# The Emerging Roles of ATP-Dependent Chromatin Remodeling Enzymes in Nucleotide Excision Repair

**DOI:** 10.3390/ijms130911954

**Published:** 2012-09-20

**Authors:** Wioletta Czaja, Peng Mao, Michael J. Smerdon

**Affiliations:** Biochemistry and Biophysics, School of Molecular Biosciences, Washington State University, Pullman, WA, USA; E-Mails: wiolettapyrzak@vetmed.wsu.edu (W.C.); pengmao@vetmed.wsu.edu (P.M.)

**Keywords:** DNA repair, DNA damage accessibility, nucleosome

## Abstract

DNA repair in eukaryotic cells takes place in the context of chromatin, where DNA, including damaged DNA, is tightly packed into nucleosomes and higher order chromatin structures. Chromatin intrinsically restricts accessibility of DNA repair proteins to the damaged DNA and impacts upon the overall rate of DNA repair. Chromatin is highly responsive to DNA damage and undergoes specific remodeling to facilitate DNA repair. How damaged DNA is accessed, repaired and restored to the original chromatin state, and how chromatin remodeling coordinates these processes *in vivo*, remains largely unknown. ATP-dependent chromatin remodelers (ACRs) are the master regulators of chromatin structure and dynamics. Conserved from yeast to humans, ACRs utilize the energy of ATP to reorganize packing of chromatin and control DNA accessibility by sliding, ejecting or restructuring nucleosomes. Several studies have demonstrated that ATP-dependent remodeling activity of ACRs plays important roles in coordination of spatio-temporal steps of different DNA repair pathways in chromatin. This review focuses on the role of ACRs in regulation of various aspects of nucleotide excision repair (NER) in the context of chromatin. We discuss current understanding of ATP-dependent chromatin remodeling by various subfamilies of remodelers and regulation of the NER pathway *in vivo*.

## 1. Chromatin Structure and DNA Accessibility

The majority of eukaryotic DNA is packaged in nucleosomes, the basic structural unit of chromatin [1]. The nucleosome consists of a core particle (~147 bp of DNA tightly wrapped around the histone octamer, composed of two H2A-H2B dimers and a H3-H4 tetramer) and the less compact DNA between nucleosome cores, called linker DNA. Nucleosomes are further assembled with linker histones and other architectural proteins, and packaged into higher order chromatin structures [2,3]. Genome-wide nucleosome organization is not random, but rather assembled into distinct arrays representing different structural and functional chromatin states. Transcriptional activity of chromatin creates specific patterns of nucleosome occupancy. It has been found that the promoter regions of actively transcribed genes are typically depleted of nucleosomes, with nucleosome occupancy progressively increasing into the coding regions [4,5].

Chromatin is an assembly that is inhibitory to cellular processes requiring direct interactions with DNA. The DNA within a nucleosome has more than 140 atomic interactions with the histone octamer, making the nucleosome core particle a stable complex, in which DNA is highly inaccessible to other DNA binding proteins [1,6]. The sterically occluding nature of nucleosomes presents a barrier to all DNA-templated processes, such as replication, transcription and repair, which all rely on the access and binding of specific proteins to their target DNA sequences. Chromatin in most eukaryotic cells is found in two distinct structural and functional forms, euchromatin and heterochromatin. Heterochromatin represents a higher level of compaction, with limited DNA accessibility and is usually associated with transcriptionally silent genes or domains. Euchromatin is decondensed, more accessible and generally associated with active transcription [7]. *In vivo*, chromatin structure undergoes dynamic remodeling, and DNA accessibility is tightly regulated by various cellular processes, including dynamic interplay between ATP-dependent complexes, covalent histone modifications, utilization of histone variants and DNA methylation.

ACRs are multi-subunit complexes containing a central DNA-dependent ATPase subunit similar to the SNF2-like family of ATPases, along with several accessory subunits [8]. The ATPase domain is related to known DNA translocases, belonging to the DEAD/H helicase family. However, unlike typical helicases, most ACRs do not have the ability to separate two annealed DNA strands [9,10]. In eukaryotes there are four distinct subfamilies of ACR enzymes, classified based on the homology between their central ATPase subunits; SWI/SNF (switching defective/sucrose nonfermenting, ISWI (imitation switch), CHD (Chromodomain, helicase, DNA binding) and INO80 (inositol requiring protein 80) [8]. ACRs are functionally diverse and play distinct roles in chromatin remodeling. The ATPase activity of remodeling complexes is stimulated by binding to their substrates, which include nucleosomes or naked DNA. A variety of studies indicate that ACRs use the energy of ATP hydrolysis to move along the minor groove of dsDNA [9]. Remodeling complexes utilize this energy to alter, break and/or re-establish DNA-histone interactions and to translocate nucleosomal DNA. This results in the ability to slide, eject and reorganize nucleosome structure and composition. Usually, the central ATPase subunit alone is sufficient to perform specific chromatin remodeling. ACRs have the ability to create and refine specific chromatin structures by ordering and phasing nucleosomes in a particular region, acting as master regulators of nucleosome positioning and occupancy in chromatin. Recent studies in yeast have reported the presence of a strongly positioned regulatory nucleosome complex, RSC/nucleosome at the *GAL*1/10 promoter upstream activating sequence (UAS), which arranges adjacent nucleosomes in specific patterns [11,12]. Indeed, other studies suggest that ACR enzymes are involved in directing the positioning of the majority of nucleosomes within the *Saccharomyces cerevisiae* genome [4].

ATP-dependent chromatin remodeling complexes regulate a wide variety of cellular processes including transcription, replication, cellular differentiation, DNA damage response and DNA repair. Given the master role of ACRs in chromatin and genome stability, there is also increasing evidence connecting ACRs to cancer. Mutations in SWI/SNF subunits (SNF5, BRG1, BRM) have been detected in several types of human cancers [13,14]. However, it remains unclear whether the tumor suppressor functions of ACRs require ACR-mediated chromatin remodeling [13]. Several recent reviews discuss various aspects of chromatin remodeling and NER [15–20]. This review will specifically focus on the role of ACRs enzymes in regulation of NER in chromatin.

Chromatin structure, even though highly compacted, does not protect DNA against the multitude of genotoxic agents to which it is exposed [21]. Damaged DNA, if left unrepaired, alters genome and epigenome stability and is a driving force in cancer development and aging. DNA repair pathways must operate in the context of chromatin, which generally restricts accessibility of DNA repair proteins to damaged DNA. Over three decades ago, Smerdon and Lieberman demonstrated that chromatin undergoes significant structural rearrangements during NER [22]. Despite significant advances in the field of chromatin biology since that time, the molecular mechanisms of the chromatin rearrangements during DNA repair remains largely unknown [20]. However, emerging evidence suggests that ATP-dependent chromatin-remodeling enzymes play important roles in the regulation of nucleosome structures at the site of damage, recruitment and assembly of the DNA repair machinery, checkpoint signaling and restoration of the chromatin structure after repair [16,23]. Nonetheless, the mechanistic details revealing how ACR enzymes modulate chromatin to coordinate DNA repair steps remain elusive.

## 2. Nucleotide Excision Repair (NER) in Chromatin

DNA in all organisms is constantly under assault by DNA-damaging agents from both endogenous and exogenous sources [24]. Endogenous agents, including reactive oxygen species (ROS) derived from cellular metabolism, can damage DNA bases (e.g., via oxidation). Exogenous agents, such as ultraviolet (UV) light, environmental chemicals, and ionizing radiation (IR), can modify DNA bases or cause DNA strand breaks. These “DNA lesions”, if left unrepaired, can block DNA replication or RNA transcription, thus leading to cell death [25]. Some DNA lesions are converted to mutations during DNA replication, which may significantly enhance genome instability and increase cancer risk [25]. Ultraviolet (UV) light is one the most ubiquitous environmental DNA damaging agents known. UV light primarily induces cyclobutane pyrimidine dimers (CPDs) and 6,4-photoproducts (6,4-PPs) [26] and both lesion types can significantly bend and distort the helical structure of DNA [27]. UV photoproducts, as well as a wide variety of other DNA lesions, are mainly repaired by the nucleotide excision repair (NER) system [28]. The extreme importance of NER in humans has been highlighted by the tight association between NER deficiency and three well-characterized genetic diseases, xeroderma pigmentosum (XP), Cockayne syndrome (CS) and trichothiodystrophy [25,29]. These diseases show symptoms such as markedly increased skin cancer risk, premature aging, developmental abnormalities, and neuro-degeneration.

There are two subpathways in NER, differing only in the damage recognition step [30]. Transcription-coupled nucleotide excision repair (TC-NER) is initiated by a lesion-stalled RNA polymerase and focuses on repair in the transcribed strand of actively transcribed genes (for review, see reference [31]). The human Cockayne syndrome (CS) protein CSB is essential for the initiation of TC-NER. CSB interacts tightly with stalled RNA Pol II and helps recruitment of factors such as transcription factor II H to the transcription bubble [32]. Another protein, UV-sensitivity scaffold protein A (UVSSA), has also been implicated in TC-NER by recruiting USP7, a ubiquitin-specific protease, to stabilize the CSB-RNA Pol II complex [33–35] The other subpathway of NER is global genome repair (GG-NER) and is responsible for repairing lesions throughout the genome. Unlike TC-NER, GG-NER employs several factors to monitor helix-distorting damage in DNA, including the UV-damaged DNA binding protein (DDB) [36] and XPC-HR23B complexes [37]. After recognition of DNA damage, both subpathways recruit transcription factor IIH (TFIIH) to damaged sites to unwind the DNA [38]. Two DNA endonucleases, ERCC1/XPF and XPG then make incisions 5′ and 3′ to the damaged site, respectively. This dual incision results in the removal of a DNA fragment, 24–32 nt long in human cells, containing the damage [39]. Subsequently, a DNA polymerase is recruited to synthesize DNA using the undamaged strand as template to fill the gap. Finally, DNA ligase functions in sealing the nick [28]. Although NER factors have been identified and characterized extensively by combined genetic and biochemical analyses, their function in the context of chromatin is still elusive. Numerous studies have demonstrated that nucleosome structure serves as a barrier to NER machinery and UV lesions in nucleosomal DNA are repaired at a significantly slower rate than in naked DNA [21,40]. For example, recognition of CPDs by XPC-HR23B is largely inhibited by the presence of a nucleosome *in vitro* [41]. The strong inhibition of NER by nucleosome structure suggests that chromatin remodeling mechanisms are necessary for the access of NER machinery to chromatin-associated DNA [40,42]. In addition to DNA repair, NER factors are involved in other cellular processes, including histone modification and transcriptional activation (for a review, see reference [43].) For instance, the mammalian UV-DDB complex has been shown to play important roles in ubiquitinating histones. Wang *et al.* demonstrated that DDB forms a ubiquitin ligase complex with CUL4 and ROC1 and ubiquitinates histone H3 and H4 [44], and this ubiquitination can facilitate the binding of XPC to UV- damaged nucleosomes. Similarly, UV-DDB also ubiquitinates histone H2A in response to UV radiation in human cells [45]. The direct role of NER factors in transcription in the absence of exogenous DNA damaging agents has also drawn much attention recently. Le May *et al.* reported the recruitment of NER factors to actively transcribed promoters, and their recruitments are necessary for the occurrence of DNA demethylation and histone modifications in the promoter region [46].

## 3. Modulation of NER by ATP-Dependent Chromatin Remodelers

It is well established that TC-NER in the transcribed strand of genes, during which nucleosomes are unstable or absent, is uniform and rapid [47]. Conversely, GG-NER repairing the non-transcribed strand of genes is heterogenous, slower and modulated by nucleosome positioning [40]. In addition it has been determined *in vitro*, that human NER complexes need a nucleosome-free DNA space of ~80 bp–100 bp to access and efficiently excise UV photoproducts, highlighting a requirement for transient disruption of one or more nucleosomes [48]. As nucleosome structures inhibit NER, chromatin remodeling activities might be required for efficient repair *in vivo* [49–51].

The possibility of a direct link between chromatin remodeling and the early steps of NER has been investigated for over a decade [49–51]. Initially, it was shown that purified ISWI-like complexes stimulate NER in dinucleosomes *in vitro* [51]. Since these reports appeared, a variety of chromatin remodeling complexes have been shown to play a role in promoting various DNA repair pathways. For example, at least five distinct ATP-dependent chromatin remodeling complexes (Ino80, Swr1, RSC, Swi/Snf, Chd1; representing all four ACRs subfamilies) are recruited and directly involved in distinct steps of double-strand break (DSB) repair and/or regulate DSB-damage responses in chromatin [52–58].

There are four conserved subfamilies of ACRs (SWI/SNF, ISW1, CHD1 and INO80) and their potential involvement in NER specifically is unclear. We have analyzed yeast mutants with deficiencies in the catalytic ATPase subunit in each of the four ACRs for their sensitivity to UV light, and mutants in all four ACR subfamilies exhibit only moderate UV sensitivity (W. Czaja and M. Smerdon, unpublished results). Furthermore, the UV sensitivity in two ACR double mutants is only moderately increased, suggesting that different ACRs might have partially overlapping, but also specialized, functions in NER. Studies by Gong *et al.* and Sarkar *et al.* demonstrated the requirement of SWI/SNF and INO80 complexes, respectively, for removal of CPDs from the transcriptionally silent *HML* locus in yeast [59,60].

## 4. SWI/SNF Subfamily

The SWI/SNF complex was originally purified from *Saccharomyces cerevisiae* [61]. Homologous complexes have also been identified in *Drosophila* and mammals [62,63]. SWI/SNF complexes are composed of 9 to 13 subunits. The central ATPase subunit is composed of an ATPase domain and additional flanking domains including the bromodomain, HAS (helicase-SANT) and post-HAS [14,64]. The bromodomain in particular has been shown to interact with acetylated lysine residues in the *N*-terminal tails of H3 and H4 *in vitro* [65]. In human cells there are two distinct SWI/SNF-like ATPase subunits, named hBRM (human Brahma) and BRG1 (Brahma-Related Gene 1) [66]. Biochemical studies with the SWI/SNF subfamily of remodelers demonstrated that the particular ATPase subunit specifies the remodeling outcome and is necessary for assembly of the remodeling complex [67]. Therefore, loss of the central ATPase subunit may preclude assembly of the remodeling complex and eliminate remodeling activity. In yeast cells, the SWI/SNF complex is present at relatively low abundance; ~200 molecules per cell [68]. SWI/SNF complexes play a major role in gene transcriptional regulation, chromosome stability and nuclear organization. Moreover, mutations in human SWI/SNF subunits have been linked to several types of cancer [14,69].

SWI/SNF remodelers are able to slide and eject nucleosomes and their function is often correlated with nucleosome disorganization, though the details of the remodeling mechanism are not well understood [70]. It has been proposed that the ATPase subunit of SWI/SNF complexes binds to a specific location on the nucleosome and utilizes its translocase activity to draw DNA from one side of the nucleosome and “pump” it toward the other side in the form of a directional wave [71].

A series of *in vitro* studies revealed links between the SWI/SNF remodeler and NER. Studies by Hara *et al.* provided the first indication that SWI/SNF complexes are able to stimulate NER *in vitro* [49]. In this study, mononucleosomes were reconstituted with 200 bp duplex DNA containing an acetylaminofluorene-guanine (AAF-G) adduct within the nucleosome core particle. It was shown that the yeast SWI/SNF complex increases the accessibility of DNA in the nucleosome core and stimulates incision at the AAF-G adduct by purified human excision nuclease [49]. In another report, these authors showed that the yeast SWI/SNF complex specifically enhanced incision at both the AAF-G adduct and a 6-4 photoproduct, located at (or near) the dyad axis of symmetry of the nucleosome core by human excision nuclease [50]. Surprisingly, these authors also found that the yeast SWI/SNF complex does not enhance incision at a CPD photoproduct located at the same position in the nucleosome core.

Further studies established closer connections between SWI/SNF and NER. Gaillard *et al.* showed that incubation of purified SWI/SNF complex with a UV-damaged nucleosome, altered the conformation of nucleosomal DNA and promoted repair by UV photolyase [72]. This observation suggests that chromatin remodeling complexes act on the structure and conformation of nucleosomes to stimulate repair.

**Figure 1:**
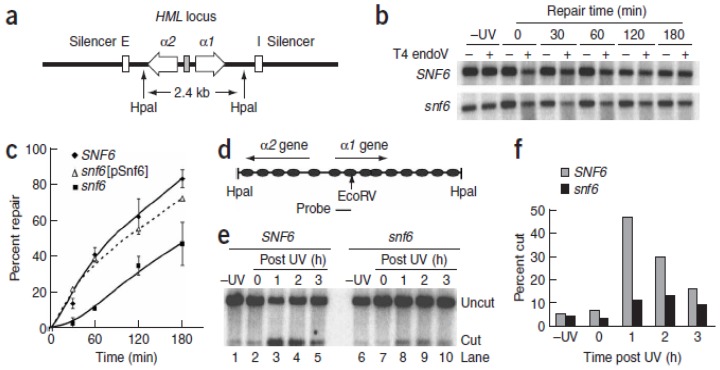
Inactivation of switching defective/Sucrose nonfermenting (SWI/SNF) affects the rate of global genome repair (GGR) at the silent *HML* locus in yeast cells. (**a**) Schematic diagram of the *HML* locus, including *a1* and *a2* mating-type genes and essential (E) and important (I) *cis*-acting silencer sequences that maintain transcription silencing; (**b**) Representative gel showing CPD removal. Yeast cells were UV-irradiated and incubated for times indicated. DNA purified from cells was assayed using the CPD-specific T4 endonuclease V (T4 endo V); (**c**) Time course for CPD removal. Data represent means ± SD from three independent experiments. *snf6* (pSnf6) strain expresses Snf6 from a plasmid; (**d**) Schematic diagram of the nucleosome-loaded *HML* locus. Ovals represent nucleosomes whose positions have been mapped at nucleotide resolution20; (**e**) Accessibility of the *Eco*RV site at the *HML* locus in chromatin of isolated nuclei after UV irradiation; (**f**) Quantitative data showing the percentage of DNA accessible to *Eco*RV.

Gong *et al.* provided direct evidence *in vivo* linking the SWI/SNF complex with NER in intact yeast cells [59]. A major finding of this report was demonstration of direct interactions between the subunit of SWI/SNF, SNF6, and the lesion recognition heterodimer Rad23-Rad4 (the ortholog of human XPC-hHR23B). The SWI/SNF interaction with RAD23-Rad4 was enhanced in a UV-dependent manner, suggesting that the remodeler might be specifically recruited to sites of damage by NER lesion recognition proteins. Furthermore, this study demonstrated a specific role of SWI/SNF during GG-NER. That is, the SWI/SNF complex is required for efficient removal of CPDs by GG-NER at the silent (nucleosome-loaded) *HML* locus (Figure 1a–c). Additionally, it was found that chromatin accessibility at *HML* was significantly decreased in SWI/SNF deficient yeast cells (Figure 1d–f). Collectively, these data suggest that SWI/SNF contributes to UV-induced chromatin remodeling, in at least certain regions of chromatin, facilitating DNA accessibility and enhancing NER *in vivo*.

A direct role of SWI/SNF in NER has also been established in mammalian cells. Two studies by the Wani lab analyzed the function of SWI/SNF in regulation of NER in mammalian cell chromatin [73,74]. Ray *et al.* demonstrated that hSNF5, a component of human SWI/SNF complex, interacts with the UV damage recognition factor XPC-hHR23B, and accumulates at sites of damage after UV irradiation. These data are consistent with the studies in yeast cells [59] and suggest a conserved mechanism of recruitment of SWI/SNF to UV lesions. Inactivation of hSNF5 did not affect recruitment of NER factors to UV damaged sites, but affected recruitment and activation of the ATM checkpoint kinase, resulting in defective H2AX and BRCA1 phosphorylation [74]. Defects in recruitment of ATM and MDC1-53BP1-BRCA1 disrupt the assembly of downstream repair factors and significantly impair NER in mammalian cells [74]. However, the recruitment and binding of SWI/SNF to damaged chromatin are not well understood. The absence of hSNF5 does not affect XPC recruitment and suggests that the remodeler is recruited to the site of damage by NER factors [73].

Gong *et al.* first reported that a human carcinoma cell line (SW13), which is deficient in the ATPase subunit of SWI/SNF (Brg1), is very sensitive to UV radiation [75]. These cells become significantly more UV resistant when Swi/Snf activity is restored by ectopic expression of Brg1. This UV sensitivity correlates well with inefficient removal of CPDs (but not 6-4PPs) and dramatic UV induced apoptosis, which is not observed in SW13 cells expressing Brg1 [75]. Similarly, Zhao *et al.* found that Brg1 is required for efficient removal of CPDs and not 6-4PPs in normal human fibroblasts with Brg1 levels knocked down with siRNA [74]. Furthermore, these authors found that Brg1 interacts with XPC and is recruited to UV-induced CPDs in a DDB2- and XPC-dependent manner. In addition, they report that bulk chromatin from human cells depleted of Brg1 exhibits decreased UV-induced chromatin relaxation, as well as impaired recruitment of downstream NER factors XPG and PCNA to UV lesions; the assembly of upstream factors DDB2 and XPC, however, remains unaffected [74]. The authors also observed that Brg1-deficient cells contribute to faster degradation of XPC, which further compromised XPG recruitment and NER efficiency. They speculate that Brg1 may protect XPC from degradation by promoting modification to XPC [74]. Collectively, the early studies in yeast and mammalian cells suggest that the chromatin remodeling function of SWI/SNF contributes to UV-induced chromatin remodeling, DNA damage accessibility, and assembly of repair and checkpoint proteins at damaged DNA sites associated with at least certain regions of chromatin.

Several studies indicate that the SWI/SNF complex might have special functions within regions of silent heterochromatin, where DNA access is significantly restricted by positioned nucleosomes and silencing proteins. Above, we discussed the requirement of SWI/SNF at the silent *HML* locus in yeast cells [59]. Sinha *et al.* reported that the SWI/SNF complex disrupts yeast silent heterochromatin by ATP-dependent eviction of Sir3p from the nucleosomal substrates *in vitro* [57]. Unexpectedly, none of the other ATP-dependent remodelers tested, (including RSC, INO80, SWR1 and Rad54) were able to displace Sir3p, suggesting that SWI/SNF might have specialized functions in regulating DNA accessibility at silent heterochromatin regions [57].

## 5. ISWI Subfamily

The ISWI group of enzymes was originally discovered in *Drosophila* [76]. Homologs of ISWI have been identified in plants, yeast, mice and humans. ISWI remodelers are characterized by the presence of a SANT domain in the catalytic subunit. The SANT domain has a central role in chromatin remodeling by functioning as a unique histone-tail-binding module [77]. The ISWI ATPase domain interacts with nucleosomal DNA, that is ~20 bp away from the dyad [78]. The *C*-terminal domains, including HAND, SANT and SLIDE, make contacts with the H4 histone tail and linker DNA, and mediate interactions with nucleosomal DNA and the histone core [79,80]. The ATPase and *C*-terminal domains of the enzymatic subunit are essential for ISWI chromatin remodeling. *In vitro* studies have demonstrated that the ISWI subfamily of remodelers uses ATP hydrolysis to promote chromatin assembly, deposit, slide and organize nucleosomes, and regulate nuclosome spacing [81]. In yeast cells, the ISWI complex is abundant (~1500 molecules per cell), but not essential for cell survival [68]. In higher eukaryotes, ISWI is an abundant and ubiquitously expressed protein that is essential for cell viability [82,83]. Furthermore, in yeast ISW1a complex is able to position nucleosomes at set distance from each other to create nucleosomal arrays with a uniform spacing of ~175 bp [84]. Nucleosome directional sliding by yeast ISWI has also been demonstrated in intact cells [85]. ISWI complexes play a central role in chromatin compaction by promoting the association of linker histone H1 [86], and are able to both activate and repress transcription [83]. Recent studies by Erdel *et al.* provide new insights into the target location and translocation mechanisms for ISWI remodelers [79]. ISWI chromatin remodelers are very mobile in the nucleus and these authors propose that these ACRs sample nucleosomes along the DNA in transient binding reactions. These authors also demonstrated that mammalian ISWI proteins accumulate at UV-induced DNA damage sites within seconds after irradiation, suggesting that this subfamily of remodelers play a role in NER [79].

The role of ISWI remodelers in NER has not been well established. Two independent *in vitro* studies linked theISW1 class of remodelers with NER in the context of chromatin [51,72]. The first of these studies demonstrated that the *Drosophila* ISWI-like complex, ACF facilitates excision of 6-4PPs from linker regions in reconstituted dinucleosomes, by purified human NER factors [51]. The second study showed that yeast ISW2 was able to move a UV-damaged nucleosome to amore central position, and this repositioning affected the repair pattern [72]. *In vivo*, the ISW1 class of remodelers have been implicated in UV induced DNA damage response of *Caenorhabditis elegans*. Knockdown of four proteins of ISWI complex in *C. elegans* larvae resulted in increased UV sensitivity, whereas mutants in other remodelers such as INO80 or CHD were similar to wild type, suggesting specific involvement of ISWI remodelers in the DNA damage response of nematodes [87].

## 6. INO80 Subfamily

Like SWI/SNF, the INO80 chromatin remodeling complex was originally purified and characterized in yeast cells [88]. The INO80 complex contains a central ATPase subunit (Ino80) and 13–16 accessory subunits (varying among different species) [64]. In yeast cells, the INO80 complex is present at relatively high abundance; ~6200 molecules per cell [68]. INO80 complexes regulate the genome-wide distribution of histone variant H2A.Z, as it has the ability to replace nucleosomal H2A.Z/H2B with free H2A/H2B dimers [89]. *In vitro* studies with mononucleosomes revealed that the human INO80 complex catalyzes nucleosome sliding [90]. Another study with fission yeast demonstrated that the INO80 complex mediates nucleosome eviction *in vivo* [91]. The INO80 complex appears to have quite diverse cellular functions in the cell, as it regulates transcription, DBS repair, DNA damage checkpoint response and DNA replication [53,92–94].

The functional connection between INO80-dependent chromatin remodeling and NER has been demonstrated in yeast and mammalian cells [60,95]. In yeast, it was shown that the Ino80 ATPase has direct UV- inducible interactions with the Rad4-Rad23 heterodimer, and is recruited to damaged DNA in a Rad4-dependent manner [60]. However, loss of INO80 did not affect the genome-wide removal of CPDs or 6-4PPs [60,96].

INO80 has been shown to facilitate repair of CPDs at the nucleosome loaded, transcriptionally silent *HML* locus [60]. Though NER is associated with significant changes in chromatin structure, it is not well understood if these changes are the result of nucleosome sliding, eviction or other nucleosome rearrangements. Immediately after UV irradiation histone H3 loss is observed at the *HML* locus, and after DNA repair the histone occupancy is gradually restored [60]. Intriguingly, INO80 has been observed to contribute to restoration of nucleosome occupancy at *HML*, which provides a direct mechanistic link between remodeling action and chromatin rearrangements during/after NER [60]. Studies in mammalian cells have demonstrated that INO80 is important for damage binding and subsequent assembly of NER factors [95]. The INO80 complex forms UV-dependent interactions with DDB1/DDB2, but not with XPC, XPA or XPD, and mammalian INO80 facilitates removal of both CPDs and 6-4PP genome-wide. Importantly, loss of INO80 in mammalian cells has no significant impact on the transcriptional expression of the NER factors [95]. Collectively, these studies reveal that the INO80 complex is involved in various aspects of NER in chromatin.

## 7. CSB (Cockayne Syndrome Group B)

Human CSB protein (yeast ortholog, Rad26) belongs to the ERCC6 subfamily of SNF2ATP-dependent chromatin remodelers and displays a DNA-stimulated ATPase activity [97,98]. Unlike other SNF2 remodelers, CSB does not assemble into multisubunit complexes. Mutations in CSB lead to Cockayne syndrome, a disorder associated with sun hypersensitivity, neurodevelopmental abnormalities, premature aging and death [99]. Human CSB and yeast Rad26p are essential proteins for transcription-coupled repair at actively transcribed genes, however very little is known how these ATPases contribute to efficient TC-NER [47,100]. In TC-NER, the transcription elongation complex stalls at the site of a DNA lesion and RNAP II is displaced to facilitate assembly of NER repair factors [47]. Several studies demonstrated that CSB interacts with RNAP II [98,101]. *In vitro* studies by Citterio *et al.* demonstrated that purified CSB is able to bind naked DNA and change the DNA topology by inducing negative supercoiling [102]. This study also revealed that CSB protein is able to remodel nucleosome core particles making them more accessible to nucleases and reposition nucleosomes on plasmid DNA in a similar fashion to hSWI/SNF [102]. Interestingly, unlike human or yeast SWI/SNF complexes, interactions of CSB with histone tails are important for the remodeling activity [102]. The chromatin remodeling activity of CSB suggest potential involvement of CSB in chromatin rearrangements at repair sites to facilitate displacement of the stalled RNAPII complex to facilitate accessibility of NER enzymes to DNA damage [102]. Moreover, ATP hydrolysis by CSB is required for stable CSB-chromatin association following UV irradiation [103]. Collectively, ATP-dependent remodeling activity of CSB protein is important for multiple aspects of TC-NER, including lesion recognition and assembly of the repair complex.

## 8. Crosstalk between ACR and Histone Modifications

In addition to ATP-dependent chromatin remodeling, posttranslational modifications of histones are another important mechanism that can modulate the chromatin landscape. Histones have been found to be acetylated, methylated, phosphorylated, ubiquitinated, sumoylated, and ribosylated, with different modifications playing different roles in cellular processes [104]. Recent studies have demonstrated that some histone modifications may directly alter nucleosome, or chromatin, stability and assembly. For example, attachment of the relatively bulky ubiquitin moiety to H2B lysine123 has been shown to stabilize chromatin via preventing the constant eviction of H2A-H2B dimer from nucleosomes in yeast [105]. The stabilizing effect of H2B ubiquitination is also important for nucleosome (re)assembly across the genome [106]. Another modification, H4K16 acetylation, which occurs on the *N*-terminal tail of histone H4, has been shown to directly inhibit the compaction of nucleosome arrays [107]. Some histone modifications may not alter chromatin structure directly, but rather act as marks to recruit effector proteins. Indeed, many protein domains can recognize modified histone residues [108]. These domains include the bromodomains that specifically recognize acetylated lysine residues [109], chromodomains and plant homeodomain (PHD) fingers that recognize methylated lysine residues [108]. When such domains are present in chromatin remodeling complexes, crosstalk between histone modifications and chromatin remodeling can occur. Previous studies have shown that the crosstalk between histone modifications and chromatin remodeling plays important roles during transcription regulation. For instance, Hassan *et al.* used a purified system to demonstrate that SWI/SNF binding to an unmodified promoter nucleosome array is unstable and its retention requires the presence of a transcription activator [110]. However, when nucleosomes are acetylated by the SAGA or NuA4 histone acetyltransferase (HAT) complexes, the stability of SWI/SNF binding is increased and the binding is maintained after disassociation of the transcription activator. In addition, these authors found that the bromodomain in the Snf2 subunit of SWI/SNF is responsible for recognizing histone acetylation, and SWI/SNF lacking this domain cannot be retained on acetylated nucleosome arrays when the transcription activator is removed [111].

Nag *et al.* found that the highly modified *N*-terminal tail of H2B might be involved in binding of the SWI/SNF complex during NER, implicating a role for histone posttranslational modification on H2B in potential recruitment and binding of the ACR enzyme [112]. Moreover, the Rsc4 subunit of the RSC remodeler contains tandem bromodomains which specifically recognize acetylated H3 at lysine 14 *in vitro* [113]. Mutation of *Rsc4* together with *Gcn5*, which encodes the HAT for H3K14, leads to cell lethality [113]. Interestingly, H3K9 and K14 are hyperacetylated following UV irradiation, and Gcn5 is important for UV-induced H3 acetylation at K9 and K14 and NER in the *MFA2* gene locus [114]. H3 acetylation stimulated by UV does not directly affect chromatin accessibility [114], but more likely acts as a signal to recruit chromatin remodeler(s) such as RSC or SWI/SNF. On the other hand, UV-induced H3 acetylation is dependent on Rad16 [115], a GG-NER factor. Intriguingly, Rad16 is very homologous to Snf2 [116], the ATPase catalytic subunit of SWI/SNF. Furthermore, biochemical studies have shown that Rad16 can generate superhelical torsion in DNA [117], a common function among SWI/SNF-like proteins. The Rad16-mediated H3 acetylation suggests that chromatin remodeling may play important roles in establishing histone acetylation following UV irradiation.

In addition to NER, several recent studies have highlighted the crosstalk between histone modifications and chromatin remodelers in the repair of double strand breaks (DSBs). For instance, mammalian SWI/SNF has been shown to bind to nucleosomes containing phosphorylated H2AX (γ-H2AX) in response to ionizing irradiation (IR), and the binding is dependent on the phosphorylation site [118]. Surprisingly, SWI/SNF does not contain protein domains that recognize phosphorylated amino acid residues. It turns out that γ-H2AX is essential for the IR-induced H3 acetylation in nucleosomes bearing γ-H2AX and acetylated histones act as a signal to recruit BRG1 of SWI/SNF via its bromodomain [118]. Furthermore, the binding of the INO80 complex to DSB sites is dependent on its recognition of the damage-induced γ-H2AX, and mutation of either the kinases that phosphorylate H2AX or the phosphorylation residue on H2AX significantly reduces the recruitment of INO80 to DSBs [58,119]. These studies have clearly shown the essential roles of γ-H2AX in recruiting chromatin remodeling complexes to facilitate repair of DSBs.

## 9. Concluding Remarks and Perspectives

**Figure 2:**
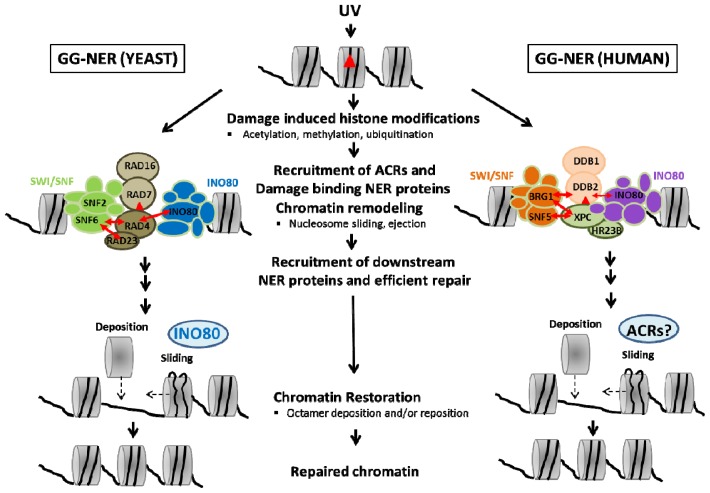
Model demonstrating involvement of ATP-dependent chromatin remodelers (ACRs) in Global Genome Nucleotide Excision Repair (GG-NER). UV DNA lesions (red triangle) induce variety of histone modifications such as acetylation, methylation, ubiquitination. These modifications serve as signals for recruitment of ACRs and damage binding NER proteins to the damage site. The early Nucleotide Excision Repair (NER) proteins (Rad7/Rad16 and Rad4-23, DDB2/DDB1 and XPC, HR23B) arrive at the damage site, recognize and specifically bind UV lesions. Chromatin remodelers, SWI/SNF and INO80 are recruited by histone modifications and/or interactions with damage binding proteins (red double head arrows). Yeast and human ACRs are distinguished by color coding. Yeast SWI/SNF (light green), human SWI/SNF (orange), yeast INO80 (dark blue), human INO80 (purple). Remodelers catalyze chromatin remodeling (nucleosome sliding, ejection), facilitate accessibility of damaged DNA and help recruitment and assembly of the downstream NER repair proteins. The UV lesions are efficiently removed. Chromatin structure is restored to the original state potentially by nucleosome rearrangements such as octamer sliding or deposition which involves action of ACR.

Evolutionarily conserved ATP-dependent chromatin remodeling complexes have emerged as master regulators of DNA repair pathways in chromatin. ACRs facilitate GG-NER in chromatin in both yeast and human systems. ACRs remodel damaged chromatin, facilitate accessibility of damaged DNA, help recruitment and sequential assembly of NER proteins and contribute to restoration of chromatin structure. Recent studies have provided significant results directly linking ACRs with chromatin remodeling during NER (Figure 2, Table 1). Further studies are necessary to delineate more of the mechanistic details that integrate changes in chromatin structure and dynamics with efficient DNA repair.

## Figures and Tables

**Table 1:** ATP-dependent chromatin remodeling enzymes involved in NER.

ACR complex (Number of subunits)	ACR abundance (Molecules per cell)	DNA-dependent ATPase subunit in Yeast and human ACR complexes		Chromatin remodeling activity of ACR complex	Role of ACR in NER
				
		Yeast	Human		
SWI/SNF (9–13) [14,64]	SWI/SNF (~200) [68]	Swi2/Snf2	BRG1, hBRM	Nucleosome sliding, displacement *in trans* [70]	Enhances NER in yeast and mammalian cells [59,73,74]
ISWI (2–5) [64]	ISWI (~1500) [68]	Isw1	hSNF2L	Nucleosome sliding, spacing and assembly [79,84]. Promotes chromatin compaction and H1 association with linker [86]	Facilitates NER in nucleosomes *in vitro* [51] Implicated in UV DNA damage response [87]
INO80 (13–16) [64]	INO80 (~6000) [68]	Ino80	hIno80	Nucleosome sliding [90] and eviction [91]	Enhances NER in yeast cells [60] Promotes NER in mammalian cells [95]
CSB (1) [103]	-	-	CSB	Nucleosome core remodeling, nucleosome repositioning [102]	Enhances TC-NER in mammalian cells [47,103]
Rad26 (1) [100]	Rad26 (<50) [68]	Rad26	-	-	Stimulates TC-NER in yeast cells [100]

## Abbreviations

ACR: ATP-dependent chromatin remodeler
ATP: Adenosine-5′-triphosphate
BRG1: Brm-related gene 1
BRM: Brahma protein
CHD: Chromodomain, Helicase, DNA binding
CPD: Cyclobutane pyrimidine dimer
DEAD/H: Asp-Glu-Ala-Asp/His helicases
ERCC1: Excision Repair Cross Complementing
GG-NER: Global Genome Nucleotide Excision Repair
HML: Hidden Mat Left
ISWI: Imitation Switch
INO80: Inositol Requiring 80
NER: Nucleotide Excision Repair
PHD: Plant Homeo Domain
RSC: Remodel the Structure of Chromatin
SANT: DNA binding domain in SWI3, ADA2, N-CoR and TFIIIB
SLIDE: SANT like ISWI
SNF2: Sucrose Non-Fermenting
SWI/SNF: Switching defective/Sucrose nonfermenting
TC-NER: Transcription Coupled Nucleotide Excision Repair
XPC: Xeroderma Pigmentosum Group C
XPF: Xeroderma Pigmentosum Group F
XPG: Xeroderma Pigmentosum Group G
